# The Efficacy of Vectron 20 WP, Etofenprox, for Indoor Residual Spraying in Areas of High Vector Resistance to Pyrethroids and Organochlorines in Zambia

**DOI:** 10.5402/2013/371934

**Published:** 2012-09-05

**Authors:** Emmanuel Chanda, Alister Kandyata, Javan Chanda, Faustina N. Phiri, Lucy Muzia, Mulakwa Kamuliwo

**Affiliations:** ^1^National Malaria Control Centre, Ministry of Health, Directorate of Public Health and Research, P.O. Box 32509, Chainama College Grounds, 10101 Lusaka, Zambia; ^2^Zambia Integrated Systems Strengthening Programme, Malaria Control Unit, Plot 1321 Enock Kavu Road, Rhodes Park, P.O. Box 39090, 10101 Lusaka, Zambia

## Abstract

The selection of insecticide resistance in malaria vectors has the potential to compromise any insecticide-based vector control programme. To ensure that the insecticides used for indoor residual spraying and insecticide-treated nets in Zambia remain effective and their choice is evidence based, insecticide resistance surveillance and monitoring are essential. This study assessed and compared the residual efficacy of etofenprox (Vectron 20 WP), an ether pyrethroid, at 0.1 g/m^2^ with pyrethroids: bifenthrin (Bistar 10 WP) and lambda-cyhalothrin (Icon 10 CS) at 25 mg/m^2^ for indoor residual spraying. We also assessed the resistance status of etofenprox to local malaria vectors, *An. funestus* s.s and *An. gambiae* s.s, using World Health Organization standard protocols. The residual efficacy of Vectron 20 WP on cement, rendered walls of houses lasted for four months with 100% mortality. By the eighth month, the killing effect had reduced to 73.8% compared to 63.3% for bifenthrin and 77.0% for lambda-cyhalothrin. Susceptibility tests using standard World Health Organization assays on *An. gambiae* s.s showed susceptibility to etofenprox (0.1%) but some resistance was detected to *Anopheles funestus* s.s. The product is recommended as an ideal insecticide for indoor residual spraying for malaria control in Zambia as part of a resistance management programme in selected areas of the country.

## 1. Introduction

Globally, about 515 million malaria cases with over one million deaths occur annually in tropical and subtropical regions [1]. The greatest toll is exacted in sub-Saharan Africa, particularly in children and pregnant women [2, 3]. This huge burden of disease is as a consequence of the excellent vectorial competence of the three major vectors of malaria: *Anopheles gambiae* s.s, *Anopheles arabiensis,* and *Anopheles funestus* [4, 5]. Malaria remains a major public health challenge and continues to severely undermine the socioeconomic growth in sub-Saharan Africa [6]. In the absence of a vaccine, much of the malaria control efforts rely primarily on effective treatment with antimalaria drugs and prevention through transmission-blocking vector control interventions [7, 8]. Nevertheless, effectiveness of malaria control is threatened by the increasing levels of both drug resistance in *Plasmodium *parasites [9] and insecticide resistance in Anopheles vectors [10]. 

The main thrust contemporary vector control interventions, that is, indoor residual spraying (IRS) and insecticide treated nets (ITNs), are insecticide based [11]. However, the arsenal of insecticides is so limited with only four classes being available for control. Presently there are only 12 registered insecticides for IRS and 6 for ITNs [12, 13]. Moreover, indiscriminate and persistent utilization of insecticides invariably exacerbate resistance development in the vectors that they are intended to control [14–16]. Insecticide resistance in malaria vectors to pyrethroids and dichloro-dimethyl-trichloroethane (DDT) has been reported across sub-Saharan Africa [16]. In Zambia, resistance has been detected in both *An. gambiae* s.s and *An. funestus* s.s to pyrethroids and DDT [17]. There is an urgent need for the identification of alternative effective insecticides for vector control. 

In this light, Vectron 20 WP was evaluated for potential use for IRS in the national malaria control programme in Zambia. Etofenprox, a pyrethroid-like insecticide devoid of an ester bond, has very low mammalian toxicity and the highest safety factor [18, 19]. A few laboratory and field trials have been carried out with this insecticide in different countries [18–20]. The product is recommended for indoor residual spraying by World Health Organization Pesticide Evaluation Scheme (WHOPES) at 0.1–0.3 g/m^2^ with an effective duration for 3–6 months [18]. 

This study evaluated the persistence of the biological efficacy of Vectron 20 WP IRS against *Anopheles gambiae* s. s and *An. funestus* s.s (Diptera: Culicidae) in areas of high insecticide resistance in field tropical conditions of Zambia. 

## 2. Materials and Methods

### 2.1. Experimental Sites

Zambia is situated in the Southern African region with a population of approximately 13 million, 45% of whom are below the age of fifteen [21]. The climatic conditions in the country include three distinct seasons; warm and rainy season (November–April), cool and dry season (April–August), and a hot and dry season (August–November). In winter the temperatures fall to as low as 4°C and during summer the temperature rises to as high as 38°C and varies as a function of altitude. Residual efficacy studies were carried out at Bauleni in Lusaka district where formal structures are predominant. Mosquitoes for susceptibility assays were collected from IRS operational areas of high insecticide resistance, particularly, Chipulukusu in Ndola district and Malata in Katete districts. 

### 2.2. Insecticide Dosage and Spraying

Vectron 20 WP (2-(4-ethoxyphenyl)-2-methylpropyl 3-phenoxybenzyl ether) supplied by Mitsui Chemicals Agro, Inc. Japan, and bifenthrin 10 WP (2-methylbiphenyl-3-y1methyl (Z)-(IRS)-cis-3(2-chloro-3, 3,3-trifluroprop-l-enyl)-2,2-dimethyl cyclopropane carboxylate) manufactured by FMC Corporation, USA, and Icon 10 CS (cyano-3-phenoxybenzyl-3-(2-chloro-3,3,3-trifluoroprop-1-enyl)-2,2-dimethylcyclopropane carboxylate) manufactured by Syngenta, Switzerland, were applied following manufactures instructions. 

Vectron 20 WP was applied at 0.1 g active ingredient (etofenprox) per meter square of spray surface, Bistar 10 WP and Icon 10 CS at 25 mg active ingredient (bifenthrin and lambda-cyhalothrin)/m^2^, respectively. All applications were done with the Hudson X-Pert compression spray pumps (H.D Hudson Manufacturing Company, USA) after calibration of 8001E nozzle according to standard procedure. Insecticides were mixed in 10 liters of clean water in the spray can and pressurized to 55 Psi. The spraying coincided with national malaria control programme spraying campaign and was conducted by competent spray operators trained in accordance with the standard training guidelines [22]. 

### 2.3. Cone Bioassay Test

The residual efficacy of etofenprox (Vectron 20 WP), an ether pyrethroid, at 0.1 g/m^2^ was compared with pyrethroids: bifenthrin (Bistar 10 WP) and lambda-cyhalothrin (Icon 10 CS) at 25 mg/m^2^ and control. Residual efficacy of all insecticides was determined on randomly selected sprayed cement surfaces of uniform texture. The controls (no insecticide) were conducted on untreated card boxes fixed on the wall. Contact bioassays of three replicates (15 mosquitoes per dose/surface) using WHO-supplied bioassay cones were performed 1 day after spraying, and subsequently on the same day of each month for 7 months. 

World Health Organisation standard bioassays were conducted [23, 24] using sugar-fed, 48 to 72 h old female *An. gambiae *s.s Kisumu strain from a colony maintained at the National Malaria Control Centre in Lusaka, Zambia. Three replicates of the WHO cones were adhered to the top, middle, and bottom of the surface sprayed with respective insecticides. Knockdown of mosquitoes was recorded 60 min after releasing them in cones. After exposure, mosquitoes were transferred to cups and held for 24 hrs with 10% glucose solution-soaked cotton wool before percentage mortality was calculated. The WHOPES criterion for persistence is, at least 70% mortality at 8-week period [24]. Indoor temperature and relative humidity were recorded using digital thermometer and hygrometer, respectively, each time contact bioassays were carried out.

### 2.4. Vector Susceptibility

Wild Blood-fed adult female *An. gambiae *s.l* and An. funestus *s.l were collected from houses using torches and backpack aspirators in operational areas of vector control interventions. Collected mosquitoes were transported to the laboratory and transferred to individual oviposition tubes and females allowed to lay eggs that were reared to F1 adults at 26 ± 2°C and 70–80% relative humidity. Susceptibility of sugar-fed 2–5-day-old F1 progeny was determined for DDT (4%), malathion (5%), lambda-cyhalothrin (0.05%), bendiocarb 0.1%, deltamethrin (0.05%), and etofenprox (0.1%) using the standard WHO tube method [23]. All papers were supplied by WHO. 

### 2.5. Mosquito Species Identification

Anopheline mosquitoes were identified morphologically as *Anopheles gambiae *complex*, An. funestus *group [25, 26]. Sibling species were identified using PCR [27, 28] at Malaria Institute at Macha (MIAM).

### 2.6. Statistics

The residual efficacy data on Vectron 20 WP was compared with that of conventional pyrethroids, Bistar 10 WP and Icon 10 CS and control. The significance of the differences in the mosquito mortalities was analyzed by chi-square test and odds ratio to assess the efficacy of the given formulation of insecticide. 

## 3. Results

### 3.1. Cone Bioassay Test

**Figure 1:**
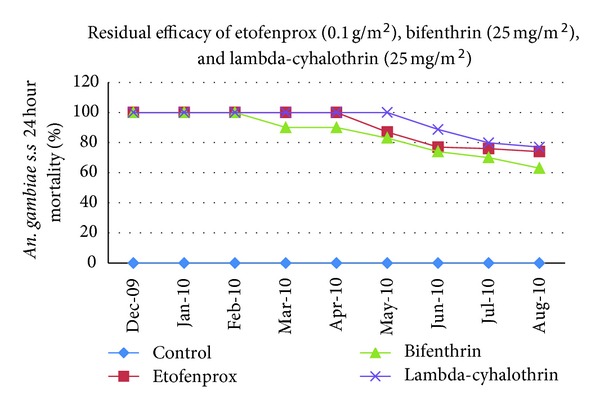
Eight months residual efficacy of etofenprox (0.1 g/m^2^), bifenthrin (25 mg/m^2^), and lambda-cyhalothrin (25 mg/m^2^) indoor residual spraying against *An gambiae* s.s.

The indoor residual spraying of Vectron 20 WP, Bistar 10 WP, and Icon 10 CS was conducted in Lusaka district, in December 2009. Cone bioassay tests revealed 100 per cent mortality 24 h after exposure up to 16 weeks for all insecticides before a gradual decline (Table 1, Figure 1). The residual effect of Vectron 20 WP and Bistar 10 WP (>80% mortality in cone bioassays) lasted for five months. Comparing the persistence of etofenprox (0.1 mg/m^2^) with that of bifenthrin (25 mg/m^2^) and lambda-cyhalothrin (25 mg/m^2^) on the cement wall surfaces using *An. gambiae *Kisumu strain showed no significant difference (*P* > 0.05). At 8 months the mortality reduced to 73.8% for etofenprox, 63.3% for bifenthrin, and 77.0% for lambda-cyhalothrin. The controls for the three insecticides registered no mortality throughout the study period. Readings for indoor temperature and relative humidity ranged from 22 to 26°C and 38 to 62%, respectively, in the houses and from 24 to 26.7°C and 76 to 90% in the insectary and showed significant changes at 24 hourly readings (Table 2).

### 3.2. Vector Susceptibility

Resistance to deltamethrin (47.3%), lambda- cyhalothrin (61.0%), and DDT (42.5%) was detected in *An. gambiae* s.s from Chipulukusu, but full susceptibile to bendiocarb, etofenprox, and malathion (Table 3). *Anopheles funestus *from Malata was fully susceptibility to malathion and resistant to deltamethrin (59.4%). Suspected resistance was detected to etofenprox (92.9%), bendiocarb (97.0%), lambda-cyhalothrin (95.8%), and DDT (93.9%) (Table 3).

## 4. Discussion

Insecticide resistance has developed in Zambia and now threatening the successful malaria control programme [17]. Although only a few operational field studies have been conducted with Vectron 20 WP, various laboratory and simulated field efficacy studies have been conducted [18–20] and the results of the present study corroborate these studies.

This efficacy study demonstrates that Vectron 20 WP, an ether pyrethroid, is as effective and persistent as the conventional pyrethroid insecticide, Bistar 10 WP and Icon 10 CS in contact bioassay field trials. Vectron 20 WP has been shown to have limiting factors in the field such as its short duration of activity and its tendency of leaving white stains on the sprayed surfaces [19]. Additionally, it is worth mentioning that the residual efficacy of this product may be compromised if it is applied on highly porous surfaces such as mud walls. However, results from this study demonstrate an improved residual efficacy of over five months on cement rendered walls. In Zambia, the active malaria transmission runs from November to April [29]. As such, the product would be appropriate in reducing transmission during this period. 

Although Vectron 20 WP has very minimal mammalian toxicity and has the highest safety factor [18], studies on the susceptibility status of local vectors to this ether pyrethroid and DDT are essential before it can be used as a safer alternative for indoor residual spray against malaria vectors. Earlier susceptibility tests conducted in Zambia using standard WHO diagnostic doses of insecticide impregnated papers on *An. gambiae *s.s revealed full susceptibility to DDT (4%), lambda- cyhalothrin (0.05%), and deltamethrin (0.05%) [11]. However, following intensive vector control efforts, variable levels of insecticide resistance have been detected in both *An. gambiae *s.s and *An. funestus* to pyrethroids and organochlorine (DDT). Significantly high levels of resistance have been detected in indoor residual spraying operational areas compared to insecticide treated nets localities. Cross-resistance between pyrethroids and DDT mediated by the knock down resistance (kdr) mutation has been detected in some areas of the country [17]. Plans are underway to deploy Vectron 20 WP operationally in areas with detectable susceptibility to this insecticide in Zambia.

Malaria has a country-wide endemicity in Zambia [30]. Successful implementation of effective malaria control has resulted in a shift in the epidemiology of the disease culminating in three distinct strata across the country [30]. Pesticide utilization both in agriculture on the high-value insecticide intensive crops and in public health for IRS and ITNs is on the increase in Zambia. Selection pressure in *An. gambiae *s.s in Ndola on the Copperbelt province could in large part be ascribed to the extensive IRS and ITNs programmes as well as the mining activities in the area. On the other hand, the high levels of resistance in *An. funestus *in Katete district in Eastern province could be as a result of agricultural use of insecticides on cotton and ITNs distribution, and to a lesser extent on IRS. In this study, *An. gambiae *s.s from Ndola, an area with high kdr-mediated-cross resistance between DDT and pyrethroids was fully susceptibile to etofenprox, malathion, and bendiocarb. Complete resistance to deltamethrin, lambda-cyhalothrin, and DDT was detected (Table 1). However, recent resistance surveillance results have indicated very high levels of etofenprox resistance in *An. gambiae* s.s in Kitwe on the Copperbelt and *An. funestus* s.s in Mbinga in eastern province. This could be a function of the focal nature of the insecticide resistance phenomenon. The suspected resistance of *An. funestus* s.s to etofenprox, bendiocarb, lambda-cyhalothrin, and DDT detected in Katete district indicates that products could still be used operationally but with rigorous insecticide resistance surveillance in place. 

Vectron 20 WP is as effective and persistent on cement walls as the competitive Bistar 10 WP and Icon 10 CS. The detected susceptibility of *An*. *gambiae* s.s and *An. funestus* to etofenprox, malathion, and bendiocarb provides an opportunity for establishing a rational insecticide resistance management strategy once the spatial heterogeneity and underlying mechanisms are determined. Vectron 20 WP is environmentally friendlier and more effective against the major malaria vectors *An. gambiae* s.s and *An. funestus *and could be rotated with organophosphates or carbamates. Therefore, it is recommended as an ideal insecticide for IRS for malaria control in Zambia as part of the insecticide resistance management strategy in selected areas of the country following expansive and rigorous surveillance.

## Figures and Tables

**Table 1:** Monthly 24−hour percentage mortality rates of *An. gambiae *s.s after exposure to etofenprox (Vectron 20 WP), bifenthrin (Bistar 10 WP) and lambda-cyhalothrin (Icon 10 CS) sprayed surfaces.

Month	[95% CI]			OR* [95% CI]	P*	[95% CI]	OR^a^ [95% CI]	*P* ^a^
	Control	Vectron 20 WP	Bistar 10 WP			Icon 10 CS	
Dec-09	0 (30)[*⋯*−*⋯*]	100 (30)[*⋯*−*⋯*]	100 (28) [*⋯*−*⋯*]	[*⋯*−*⋯*]	1	100 (30) [*⋯*−*⋯*]	[*⋯*−*⋯*]	1
Jan-10	0 (30) [*⋯*−*⋯*]	100 (30) [*⋯*−*⋯*]	100 (34) [*⋯*−*⋯*]	[*⋯*−*⋯*]	1	100 (30) [*⋯*−*⋯*]	[*⋯*−*⋯*]	1
Feb-10	0 (30) [*⋯*−*⋯*]	100 (45) [*⋯*−*⋯*]	100 (45) [*⋯*−*⋯*]	[*⋯*−*⋯*]	1	100 (41) [*⋯*−*⋯*]	[*⋯*−*⋯*]	1
Mar-10	0 (25) [*⋯*−*⋯*]	100 (35) [*⋯*−*⋯*]	90 (30) [79.26−100.74]	[*⋯*−*⋯*]	0.468	100 (35) [*⋯*−*⋯*]	[*⋯*−*⋯*]	1
Apr-10	0 (30) [*⋯*−*⋯*]	100 (43) [*⋯*−*⋯*]	90 (30) [79.26−100.74]	[*⋯*−*⋯*]	0.468	100 (40) [*⋯*−*⋯*]	[*⋯*−*⋯*]	1
May-10	0 (25) [*⋯*−*⋯*]	86.7 (30) [74.51−98.83]	83.3 (30) [69.99−96.67]	0.77 [0.19−3.20]	0.794	100 (30) [*⋯*−*⋯*]	[*⋯*−*⋯*]	0.331
Jun-10	0 (25) [*⋯*−*⋯*]	76.7 (30) [61.54−91.80]	74.3 (35) [59.81−88.77]	0.88 [0.27−2.74]	0.845	88.7 (30) [77.33−100.01]	2.38 [0.58−9.76]	0.351
Jul-10	0 (20) [*⋯*−*⋯*]	75.8 (33) [61.14−90.38]	70.7 (41) [56.80−84.66]	0.77 [0.27−2.19]	0.673	79.8 (35) [66.39−93.03]	1.26 [0.40−3.95]	0.748
Aug-10	0 (20) [*⋯*−*⋯*]	73.8 (42) [60.51−87.11]	63.3 (30) [46.09−80.57]	0.61 [0.22−1.69]	0.37	77.0 (30) [61.94−92.06]	1.88 [0.40−3.55]	0.794

*Significance of change between Vectron 20 WP and Bistar 10 WP, *Significance of change between Vectron 20 WP and Icon 10 CS.

**Table 2:** Monthly indoor temperature and relative humidity readings during the evaluation.

Date	0 Hrs		1 Hr			24 Hrs		
	Temp	RH	Temp	RH	RH^*∗*^(*P*)	Temp	RH	RH^a^ (*P*)
Dec-09	23	54	24	58	0.705	24	78	0.037
Jan-10	24	62	24.7	55	0.517	24.8	77	0.203
Feb-10	22	48	23	53	0.619	26	82	0.003
Mar-10	23.6	46	23.6	44	0.834	25.5	93	0.002
Apr-10	25.4	46	25	43.5	0.791	26.8	88	0.0003
May-10	23.2	38	24	42	0.655	25.9	90	0.00001
Jun-10	22	45	23	44	0.917	24.5	76	0.005
Jul-10	24	46	24	48	0.836	26.7	84	0.001
Aug-10	25	44	26	46	0.834	26	80	0.001

*Significance of RH at 1 hr, ^a^Significance of RH at 24 hrs.

**Table 3:** Insecticide susceptibility of 1–5-day-old F1 female progeny of *An. gambiae* s.s from Chipulukusu in Ndola district and *An. funestus* from Malata in Katete District in Zambia.

Insecticide	No. exposed	24 hr mortality (%)	Sensitivity status
*An. gambiae *s.s			
0.05% Deltamethrin	96	47.3	Resistant
0.05% Lambda- cyhalothrin	134	61.0	Resistant
4% DDT	118	42.5	Resistant
0.1% Etofenprox	151	98.7	Susceptible
5% Malathion	101	100	Susceptible
0.1% Bendiocarb	74	100	Susceptible
Controls	120	0	
*An. funestus*			
0.05% Deltamethrin	143	59.4	Resistant
0.05% Lambda-cyhalothrin	74	95.8	Suspected resistance
4% DDT	131	93.9	Suspected resistance
0.1% Etofenprox	130	92.9	Suspected resistance
5% Malathion	138	100	Susceptible
0.1% Bendiocarb	134	97.0	Suspected resistance
Controls	120	0	

## References

[1] Breman JG, Alilio MS, Mills A (2004). Conquering the intolerable burden of malaria: what’s new, what’s needed: a summary. *American Journal of Tropical Medicine and Hygiene*.

[2] Snow RW, Guerra CA, Noor AM, Myint HY, Hay SI (2005). The global distribution of clinical episodes of Plasmodium falciparum malaria. *Nature*.

[3] Guerra CA, Snow RW, Hay SI (2006). Mapping the global extent of malaria in 2005. *Trends in Parasitology*.

[4] Beier JC, Killen GF, Githure JI (1999). Short report: entomologic innoculation rates and Plasmodium falciparum malaria prevalence in Africa. *The American Journal of Tropical Medicine and Hygiene*.

[5] Hay SI, Rogers DJ, Toomer JF, Snow RW (2000). Annual Plasmodium falciparum entomological inoculation rates (EIR) across Africa: literature survey, Internet access and review. *Transactions of the Royal Society of Tropical Medicine and Hygiene*.

[6] Hay SI, Smith DL, Snow RW (2008). Measuring malaria endemicity from intense to interrupted transmission. *The Lancet Infectious Diseases*.

[7] Fillinger U, Kannady K, William G (2008). A tool box for operational mosquito larval control: preliminary results and early lessons from the Urban Malaria Control Programme in Dar es Salaam, Tanzania. *Malaria Journal*.

[8] Sharp BL, Ridl FC, Govender D, Kuklinski J, Kleinschmidt I (2007). Malaria vector control by indoor residual insecticide spraying on the tropical island of Bioko, Equatorial Guinea. *Malaria Journal*.

[9] WHO, Africa Malaria Report 2003, Geneva, World Health Organization.

[10] Ranson H, Abdallah H, Badolo A (2009). Insecticide resistance in Anopheles gambiae: data from the first year of a multi-country study highlight the extent of the problem. *Malaria Journal*.

[11] Chanda E, Masaninga F, Coleman M (2008). Integrated vector management: the Zambian experience. *Malaria Journal*.

[12] WHOPES a WHO recommended insecticides for indoor residual spraying against malaria vectors. http://www.who.int/malaria/cmcupload/0/000/012/604/IRSInsecticides.htm.

[13] WHOPES b WHO recommended insecticide products treatment of mosquito nets for malaria vector. http://www.who.int/whopes/en/.

[14] Collins FH, Kamau L, Ranson HA, Vulule JM (2000). Molecular entomology and prospects for malaria control. *Bulletin of the World Health Organization*.

[15] Hemingway J, Ranson H (2000). Insecticide resistance in insect vectors of human disease. *Annual Review of Entomology*.

[16] Coleman M, Hemingway J (2007). Insecticide resistance monitoring and evaluation in disease transmitting mosquitoes. *Journal of Pesticide Science*.

[17] Chanda E, Hemingway J, Kleinschmidt I (2011). Insecticide resistance and the future of malaria control in Zambia. *PLoS ONE*.

[18] WHO, Report of the third WHOPES working group meeting, Review of Deltamethrin 1% SC and 25% WT.

[19] Sreehari U, Mittal PK, Razdan RK, Dash AP, Ansari MA (2009). Impact of etofenprox (Vectron 20 WP) indoor residual spray on malaria transmission. *Indian Journal of Medical Research*.

[20] Hemingway J (1995). Efficacy of etofenprox against insecticide susceptible and resistant mosquito strains containing characterized resistance mechanisms. *Medical and Veterinary Entomology*.

[21] CSO, Central Statistical Office Zambia National Census Report 2000, 2000.

[22] MoH National Guidelines for Indoor Residual Spraying in Zambia.

[23] W.H.O. Test Procedures for Insecticide Resistance Monitoring in Malaria Vectors, Bio-efficacy and Persistence of insecticides in treated surfaces. Report of the WHO Informal Consultation.

[24] WHO Instructions for the Bioassay of Insecticidal Deposits on Wall Surfaces.

[25] Gillies MT, DeMeillon B The Anophelinae of Africa South of the Sahara.

[26] Gillies MT, Coetzee MA supplement to: The Anophelinae of Africa South of the Sahara.

[27] Koekemoer LL, Kamau L, Hunt RH, Coetzee M (2002). A cocktail polymerase chain reaction assay to identify members of the Anopheles funestus (Diptera: Culicidae) group. *American Journal of Tropical Medicine and Hygiene*.

[28] Scott JA, Brogdon WG, Collins FH (1993). Identification of single specimens of the Anopheles gambiae complex by the polymerase chain reaction. *American Journal of Tropical Medicine and Hygiene*.

[29] MoH National Malaria Situation Analysis.Ministry of Health.

[30] MoH Zambia National Malaria Programme Performance Review 2010.

